# The Oncogenic Role of *VWA8-AS1*, a Long Non-Coding RNA, in Epstein–Barr Virus-Associated Oral Squamous Cell Carcinoma: An Integrative Transcriptome and Functional Analysis

**DOI:** 10.3390/ijms252312565

**Published:** 2024-11-22

**Authors:** Sawarot Srisathaporn, Chamsai Pientong, Chukkris Heawchaiyaphum, Thawaree Nukpook, Sirinart Aromseree, Tipaya Ekalaksananan

**Affiliations:** 1 Department of Microbiology, Faculty of Medicine, Khon Kaen University, Khon Kaen 40002, Thailand; sawarot.s@kkumail.com (S.S.); chukhe@kku.ac.th (C.H.); thawaree.n@kkumail.com (T.N.); sirinar@kku.ac.th (S.A.); 2 HPV & EBV and Carcinogenesis Research Group, Khon Kaen University, Khon Kaen 40002, Thailand

**Keywords:** long non-coding RNA, oral squamous cell carcinoma, Epstein–Barr virus, *VWA8-AS1*

## Abstract

Dysregulated long non-coding RNA (lncRNA) expression is linked to various cancers and may be influenced by oncogenic Epstein–Barr virus (EBV) infection, a known and detectable risk factor in oral squamous cell carcinoma (OSCC) patients. However, research on the oncogenic role of EBV-induced lncRNAs in OSCC is limited. To identify lncRNA-associated EBV infection and OSCC carcinogenesis, the differential expression of RNA-seq datasets from paired normal adjacent and OSCC tissues, and microarray data from EBV-negative and EBV-positive SCC25 cells, were identified and selected, respectively, for interaction, functional analysis, and CCK-8 cell proliferation, wound healing, and invasion Transwell assays. In OSCC tissues, 6731 differentially expressed lncRNAs were identified when compared to normal tissues from RNA-seq datasets, with 295 linked to EBV-induced OSCC carcinogenesis from microarray datasets. The EBV-induced lncRNA *VWA8-AS1* showed significant upregulation in EBV-positive SCC25 cells and EBV-infected adjacent and OSCC tissue samples. *VWA8-AS1* potentially promotes OSCC via the lncRNA–miRNA–mRNA axis or direct protein interactions, affecting various cellular processes. Studies in OSCC cell lines revealed that elevated *VWA8-AS1* levels enhanced cell migration and invasion. This study demonstrates *VWA8-AS1*’s contribution to tumor progression and possible interactions with its targets in OSCC, offering insights for future research on functional mechanisms and therapeutic targets in EBV-associated OSCC.

## 1. Introduction

Oral squamous cell carcinoma (OSCC) is the most common subtype of oral cancer, accounting for more than 90% of such cancers. The development of OSCC is a complex process involving genetic and epigenetic alterations. Carcinogenesis initiates with the transformation of normal oral keratinocytes, triggered by exposure to multiple risk factors such as smoking, alcohol consumption, betel quid chewing, and exposure to oncogenic viruses [1,2,3,4], resulting in genomic instability. These genetic alterations can affect essential cellular pathways such as DNA repair, cell cycle regulation, differentiation, and apoptosis, ultimately progressing to cancerous states. Moreover, malignant cells release factors such as matrix metalloproteinases (*MMPs*), reactive oxygen species, and cytokines, which facilitate invasion, migration, proliferation, and survival through the modulation of adjacent stromal cells, thereby contributing to tumor advancement and metastatic spread. A common characteristic of OSCC is the presence of red lesions with pain that exhibit slightly irregular surfaces and well-defined margins [5]. The symptoms typically emerge as the condition progresses; in its early stages, OSCC usually does not cause pain. Thereby, this lack of early symptoms contributes to delays in diagnosis. The gold standard for oral cancer treatment is surgical resection. Radiotherapy and chemotherapy serve as alternative therapeutic approaches. The implementation of these treatments is determined by the histopathological TNM stage, with others considering risk factors [6]. Unfortunately, despite advancements in treatment, the 5-year survival rate for patients with OSCC has not significantly improved over time. Therefore, to understand the process of cancer development and identify effective therapeutic targets for OSCC, the underlying molecular mechanisms must be studied.

**Figure 1:**
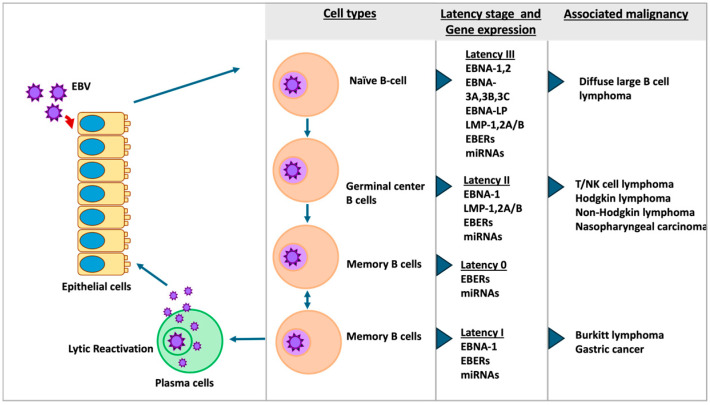
The characteristics of EBV latency stages as demonstrated by differential gene expression and associated malignancies.

The Epstein–Barr virus (EBV) is an oncogenic virus that can target both B cells and epithelial cells. Approximately 95% of individuals in the world population are asymptomatic carriers of EBV, and cancer develops in only a small minority. Following the primary infection, EBV establishes a persistent latent infection within the host. This latency is categorized into four distinct types, ranging from 0 to III (Figure 1). The virus utilizes latent infection as a mechanism to increase its progeny by stimulating the proliferation of infected host cells. This process can occasionally lead to oncogenic transformation, resulting in the development of lymphomas such as Burkitt’s lymphoma, Hodgkin’s disease, and diffuse large B-cell lymphomas [7]. Additionally, EBV can infect epithelial cells, contributing to several malignancies, including nasopharyngeal carcinoma (NPC) and EBV-associated gastric cancer [8]. While EBV-associated cancer is not predominant in the oral cavity, EBV has been identified as a potential risk factor for oral carcinogenesis [9,10]. The oral cavity often functions as a primary location for EBV presence and transmission. Research has demonstrated a positive association between EBV DNA positivity and OSCC, suggesting a connection between EBV and oral carcinogenesis [3,4]. The occurrence of EBV in OSCC differs among populations, with rates of approximately 40 to 60% [11]. Numerous studies have reported a connection between EBV and OSCC, with several EBV oncoproteins detected in OSCC cases, including EBNAs, LMP2A, 2B, EBER, LMP1, and EBNA2 [12,13,14]. The impact of EBV infection on oral cancer development has also been studied; for example, EBNA1 from EBV infection can lead to p53 inactivation [15] and LMP1 can upregulate EGFR transcription and promote the malignant transformation of epithelial cells by suppressing cancer cell apoptosis [16]. Additionally, EBV has been found to hinder epithelial cell differentiation and encourage an invasive phenotype. These characteristics persist in epithelial cells even after the loss of EBV [17]. However, some research indicates that EBV-associated cancers may require genetic alterations and could be linked to other infections like human papilloma virus, leading to debate surrounding the relationship between EBV and oral cancers [18]. Therefore, the role of EBV in OSCC carcinogenesis remains unclear.

Recently, the number of transcriptome studies in cancer research has increased. The differential expression of several coding and non-coding RNAs has been reported in oral cancers. Long non-coding RNAs (lncRNAs) are more than 200 nucleotides long and cannot be translated into proteins. They play essential roles in the regulation of various biological processes. Transcriptome profiling studies have implicated alterations in lncRNA expression in cancer development [19,20]. Multiple lncRNAs associated with oral cancer have been identified. For example, poor clinical outcomes of OSCC are related to increased expression levels of *HOTAIR*, *NEAT1*, and *UCA1* but not *MEG3*, which is expressed at lower levels [21]. Viral infections can significantly influence the expression of cellular lncRNAs [22,23]. Studies have indicated that infection with the EBV is associated with alterations in lncRNA expression patterns within cancer cells [24,25]. Investigations of the relationship between lncRNA and carcinogenesis have yielded valuable insights into the underlying mechanisms. Furthermore, some lncRNAs may be considered potential therapeutic targets. Therefore, we hypothesized that EBV may play a role in OSCC carcinogenesis by regulating host lncRNA.

In this study, we aimed to identify lncRNAs associated with the development of oral cancer in patients with OSCC and EBV infection. RNA-seq datasets of patients with OSCC from a public database and microarray data from EBV-negative and EBV-positive cultured squamous cell carcinoma (SCC) cells were used to analyze the differential expression of lncRNAs. LncRNAs overexpressed in OSCC tissues and related to EBV infection were selected to study their roles in oral carcinogenesis. The selected lncRNAs were detected in EBV-positive and EBV-negative oral cancer cells and tumor tissues and their normal adjacent tissues using quantitative reverse transcription polymerase chain reaction (qRT-PCR). The association among lncRNAs, miRNAs, and mRNAs was predicted based on the potential role of lncRNAs in binding to several types of molecules to regulate cellular processes. Functional enrichment analysis was performed to explore the molecular mechanisms of the EBV-associated lncRNAs in OSCC. For functional analysis, the effects of overexpressed lncRNAs on oral cancer cells were also studied.

## 2. Results

### 2.1. EBV-Associated lncRNAs Were Identified in OSCC Tissues

**Figure 2:**
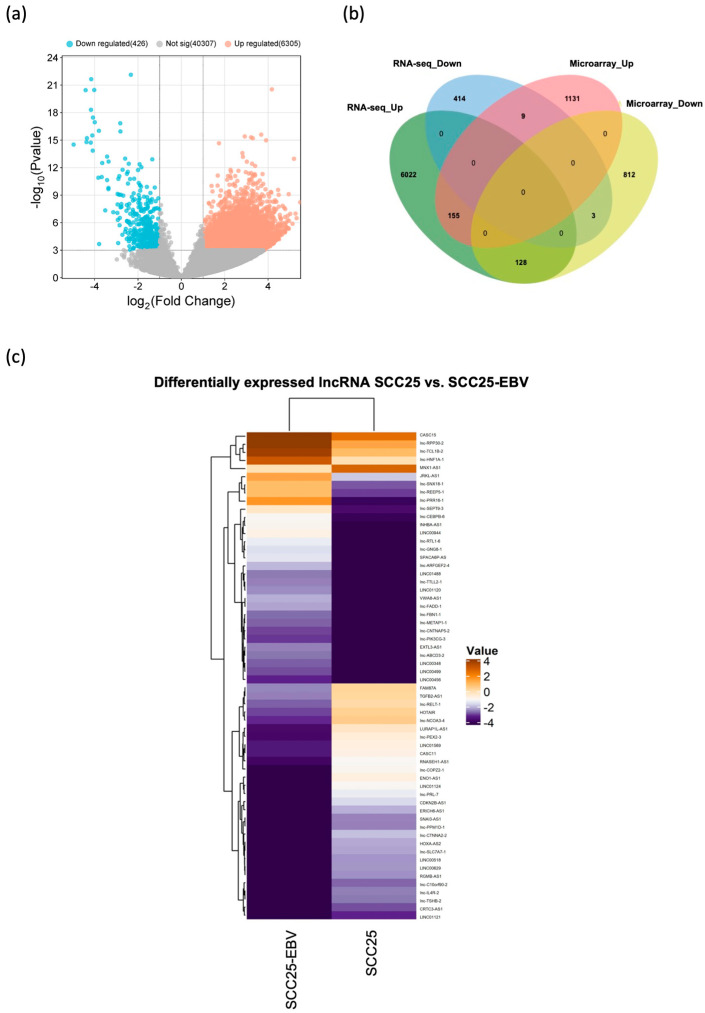
(**a**) The volcano plot illustrates the DEGs of lncRNAs in OSCC tissues relative to normal adjacent tissues from RNA-seq datasets (*p* < 0.001 and a fold-change cutoff of 2). (**b**) Venn diagram showing the lncRNAs identified via both microarray and RNA-seq analyses. (**c**) Heatmap showing the expression of 30 upregulated and downregulated lncRNAs in EBV-positive SCC25 cells compared with EBV-negative SCC25 cells from microarray datasets; the value represents the intensity of normalized data read from detected signal.

To investigate the lncRNAs associated with OSCC, we conducted a comparative analysis of lncRNA expression patterns between paired OSCC and normal adjacent tissues. This study utilized RNA-seq data obtained from the European Nucleotide Archive repository, encompassing paired tissue samples from 19 OSCC patients. The differential expression analysis of genes (DEG) revealed that a total of 6731 lncRNAs were significantly differentially expressed: 6305 were upregulated, and 426 were downregulated, in OSCC tissues (Figure 2a). This distinctive differential expression of lncRNAs in OSCC tissues suggests that alterations in lncRNA expression may play a role in OSCC carcinogenesis.

Previously, our colleagues conducted microarray data analyses using an established human tongue squamous cell carcinoma cell line (SCC25) and EBV-positive SCC25 cells [26]. A comparison between EBV-negative SCC25 and EBV-positive SCC25 (SCC25-EBV) cell lines revealed that 2238 lncRNAs were differentially expressed; 1295 were upregulated, and 943 were downregulated, in EBV-positive cells. Therefore, EBV may regulate or alter lncRNA expression in cancer cells.

Based on the RNA-seq data and microarray results, we hypothesized that EBV infection may increase the dysregulation of lncRNAs and lead to cancer development. To investigate this further, we compiled a list of differentially expressed lncRNAs via both RNA-seq data and microarray analyses. The lncRNAs identified in both the RNA-seq and microarray data were represented as EBV-associated lncRNAs. The present study showed that 295 of the 2238 lncRNAs from the microarray data were also differentially expressed via RNA-seq analysis (Figure 2b): 164 were upregulated, and 131 were downregulated, in SCC25-EBV cells compared with SCC25 cells. However, 155 lncRNAs were found to be upregulated in both RNA-seq and microarray data, while only three were downregulated in both data sources. The top 30 EBV-associated lncRNAs that displayed significant differences in the RNA-seq data of OSCC tissues and were either upregulated or downregulated in EBV-positive oral cancer cells compared with their EBV-negative counterparts, as revealed by microarray data, are shown in Figure 2c and Table 1.

### 2.2. VWA8-AS1 Overexpression Was Detected in EBV-Positive Squamous Cell Cancer Cell Lines

**Figure 3:**
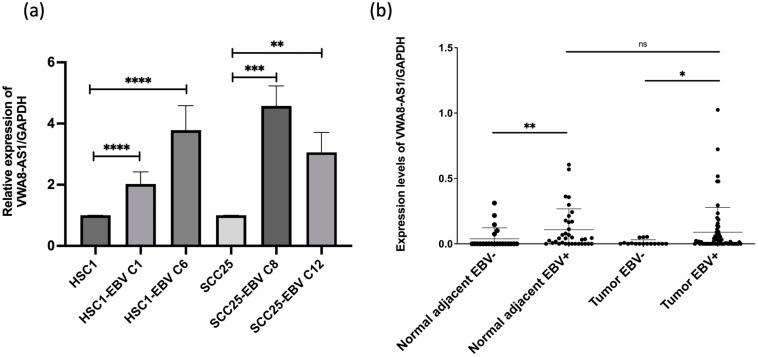
(**a**) Relative expression of *VWA8-AS1* in EBV-positive HSCs and SCC25 cells compared with that EBV-negative HSCs and SCC25 cells, respectively; Shapiro–Wilk test was used for data normality analysis (*p* > 0.05 in all groups), and the data were analyzed using *t*-tests. (**b**) Expression levels of *VWA8-AS1* in EBV-positive OSCC samples; Shapiro–Wilk test was used for data normality analysis (*p* < 0.0001 in all sample groups), and Mann–Whitney U test was used for evaluated data (* *p* < 0.05; ** *p* < 0.01; *** *p* < 0.001; **** *p* < 0.0001; ns non-significant).

To explore the expression of EBV-associated lncRNAs, we selected a lncRNA that was upregulated in OSCC tissues and SCC25-EBV cells, based on RNA sequencing and microarray analyses, respectively. The biggest fold change was shown by *lnc-PRR16-1*, followed by *VWA8-AS1*. *VWA8-AS1* is a new lncRNA not previously reported in association with OSCC; therefore, it was chosen for further study. To confirm its association between EBV and squamous cell carcinoma, two different kinds of squamous cell carcinoma with EBV infection cells were used in this study. We utilized established EBV-positive squamous cell carcinoma cell lines from our colleague’s previous research [27]. We conducted qRT-PCR to examine the expression levels of *VWA8-AS1* in different squamous cell carcinoma cell lines including EBV-negative human skin squamous cell carcinoma (HSC1) cell lines compared against the EBV-positive HSC1 clone 1 (C1) and clone 6 (C6) cell lines, respectively. Similarly, the EBV-negative SCC25 cell lines were compared against the EBV-positive SCC25 clone 8 (C8) and clone 12 (C12) cell lines, respectively. The results demonstrated that *VWA8-AS1* expression was significantly elevated in HSC1-EBV C1 and C6 compared to HSC1 (*p* < 0.0001 and *p* < 0.0001, respectively). Similar results were observed in SCC25-EBV C8 and C12 compared to SCC25 cell lines (*p* = 0.0007 and *p* = 0.0054, respectively) (Figure 3a). These results indicate that EBV infection affects *VWA8-AS1* expression.

### 2.3. VWA8-AS1 Was Overexpressed in EBV-Associated OSCC Cases

Based on EBV infection status, we conducted an additional analysis of *VWA8-AS1* expression levels in samples from normal adjacent tissues and OSCC tissues. The samples were divided into four subgroups: EBV-negative normal adjacent tissues, EBV-positive normal adjacent tissues, EBV-negative tumor tissues, and EBV-positive tumor tissues. As shown in Figure 3b, *VWA8-AS1* expression was significantly higher in EBV-positive normal adjacent tissues compared to in EBV-negative normal adjacent tissues (*p* = 0.0071), as well as in EBV-positive tumor tissues compared to EBV-negative tumor tissues (*p* = 0.0293). These findings suggest that EBV infection upregulates *VWA8-AS1* expression. Additionally, *VWA8-AS1* expression in both tumor and adjacent normal tissues suggests a potential association between EBV-induced *VWA8-AS1* and OSCC development. These findings suggest that its role in facilitating tumor progression may not be limited to the tumor site but may also extend to the surrounding EBV-infected tissue. These findings indicate that EBV can alter *VWA8-AS1* expression in a manner that is likely associated with OSCC development.

### 2.4. Predicting the Competing Endogenous (ce)RNA Role of VWA8-AS1 via lncRNA–miRNA–mRNA Interactions

The roles of lncRNAs vary, and competing endogenous RNA (ceRNA) activity is one of their functions of interest. In brief, lncRNAs can interact with miRNAs and prevent them from binding to their targets, leading to the downregulation of miRNAs and the overexpression of their mRNA targets.

**Figure 4:**
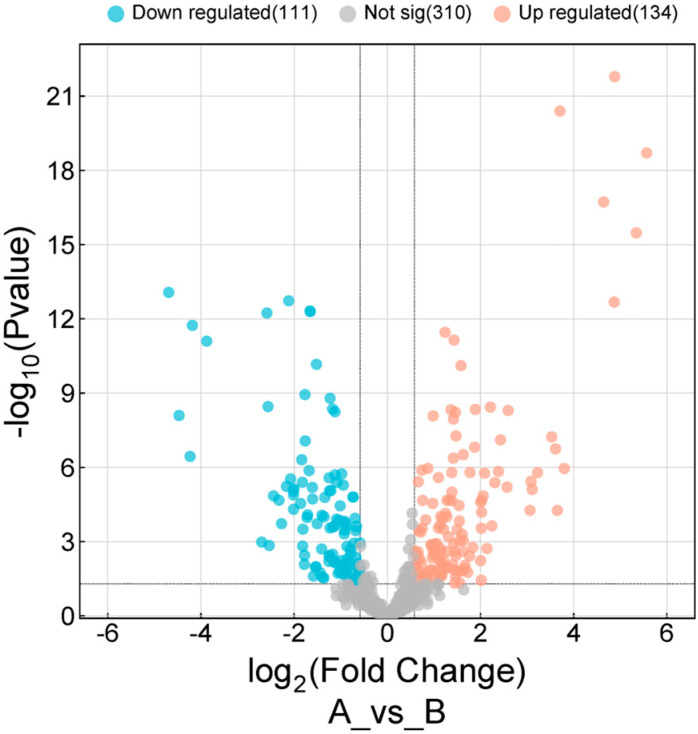
Volcano plot of fold changes in miRNA expression in OSCC relative to normal adjacent tissues (*p* < 0.05 and a fold-change cutoff of 1.5).

First, transcriptome profiling data of 16 OSCC tissues and 16 normal tissues were obtained from The Cancer Genome Atlas (TCGA) of head and neck squamous cell carcinoma (HNSCC). Then, the differential expression analysis of miRNAs in OSCC tissues compared with normal tissues was performed. One hundred and one miRNAs were downregulated, and 134 were upregulated, in OSCC tissues compared with normal adjacent tissues (Figure 4). Therefore, the dysregulation of miRNAs may be related to OSCC development.

As mentioned above, our study sought to investigate the potential interactions among lncRNAs, miRNAs, and mRNAs in OSCC and their possible associations with EBV. Given that lncRNAs can function as ceRNAs and that the expression of several miRNAs is dysregulated in OSCC, we hypothesized that EBV-associated *VWA8-AS1* may contribute to the dysregulation of miRNA expression.

To explore this hypothesis, we initially used DIANA-lncRBase to predict the interactions of *VWA8-AS1* with miRNAs. The prediction results showed the miRNAs that may be targeted by *VWA8-AS1* include hsa-miR-1-3p, hsa-miR-23b-3p, hsa-miR-125b-5p, hsa-miR-127-5p, hsa-miR-136-3p, hsa-miR-140-5p, hsa-miR-410-3p, and hsa-miR-889-3p. Notably, these miRNAs have been implicated as tumor suppressor miRNAs and are associated with a variety of cancers, such as lung [28], breast [29], cervical [30,31], gastric [32], hepatic [33], and oral cancers [34,35,36].

**Figure 5:**
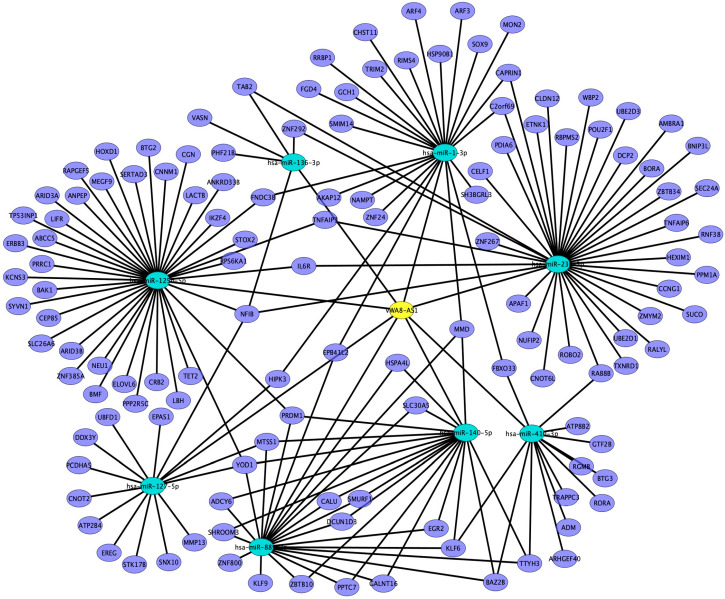
Interaction networks of *VWA8-AS1*–miRNA–mRNA (yellow: *VWA8-AS1*, blue: miRNA, purple: mRNA).

We subsequently employed four distinct databases (miRDB, miRDIP, miRTarBase, and miRTargetScan) to predict the mRNA targets of these miRNAs. We focused on mRNA targets that were identified in at least three of the databases and were found to be significantly overexpressed in the EBV-positive microarray data, as we believed these to be targets associated with *VWA8-AS1*. Based on these analyses, we constructed a comprehensive lncRNA–miRNA–mRNA network. Our findings revealed that the selected *VWA8-AS1* lncRNA targeted several common genes (Figure 5 and Table 2). Our study highlights the intricate connections among lncRNA, miRNAs, and mRNAs. We provide evidence to support the concept that EBV-associated *VWA8-AS1* can modulate miRNA and mRNA expression, thereby promoting OSCC by functioning as a ceRNA for miRNAs. However, further in vitro experiments are required to confirm the interaction in this study.

### 2.5. The Prediction of VWA8-AS1 Interactions with Proteins

**Figure 6:**
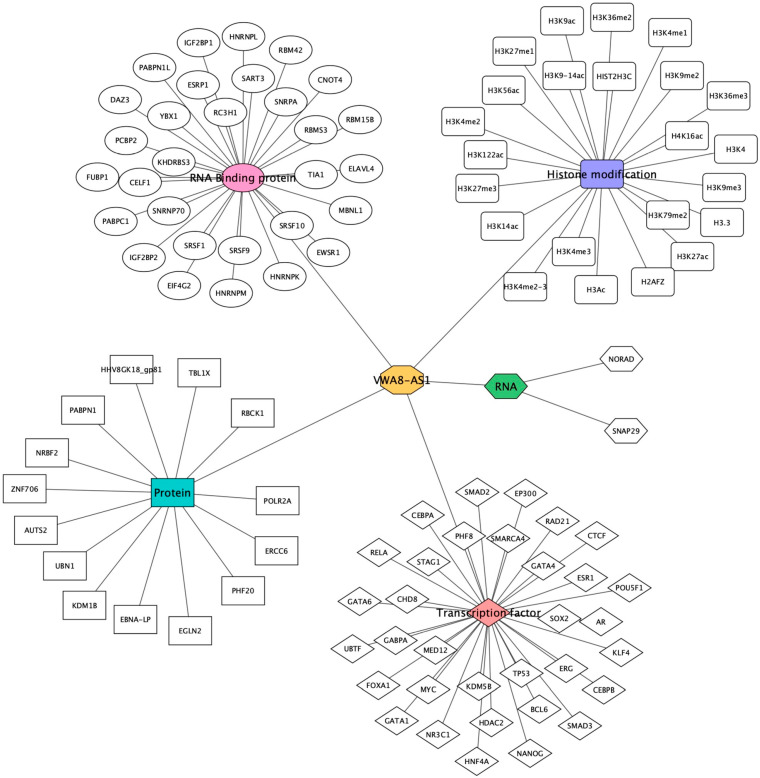
Target prediction of *VWA8-AS1*.

Another possible function of lncRNAs in regulating cellular processes is their capacity to bind with proteins. The interaction between *VWA8-AS1* and its targets was identified using RNAINTER and RBPMAP. The findings indicate that *VWA8-AS1* contains multiple binding sites for a variety of proteins, including RNA-binding proteins, transcription factors, and histone modification proteins, as well as other RNAs (Figure 6).

### 2.6. Functional Analysis of mRNA Targets Associated with VWA8-AS1

**Figure 7:**
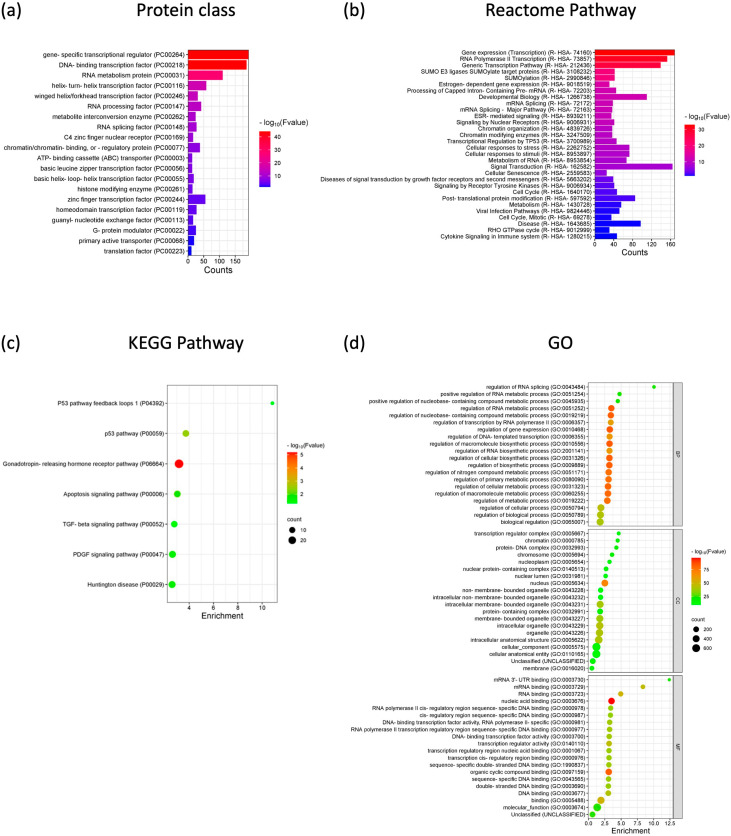
GO enrichment and KEGG pathway analysis of mRNA targets associated with *VWA8-AS1.* (**a**) Protein class. (**b**) Reactome pathway. (**c**) KEGG pathway. (**d**) GO.

Functional Gene Ontology (GO) analysis of *VWA8-AS1* was carried out by examining the predicted targets that interact with *VWA8-AS1* directly or indirectly through miRNAs, which were found to be associated primarily with gene-specific transcriptional regulators, DNA-binding transcription factors, RNA metabolism proteins, RNA processing factors, and RNA splicing factors. Additionally, a reactome pathway analysis revealed that *VWA8-AS1* is involved in several pathways, including transcription, RNA splicing, chromatin modification, post-translational protein modification, cell signaling, the cell cycle, viral infection, and cytokine signaling in the immune system. Kyoto Encyclopedia of Genes and Genomes (KEGG) pathway analysis indicated that these targets were involved in the p53, apoptosis, and TGF-β signaling pathways. Furthermore, these targets were found to be enriched in cellular components, such as the nucleus, nucleoplasm, cytoplasm, and organelles, and their functions were related to transcriptional regulatory activity, RNA and DNA binding, and the regulation of cellular, biological, and metabolic processes (Figure 7). Therefore, the prediction of *VWA8-AS1*-associated targets suggests that *VWA8-AS1* may play a role in several cellular and biological regulatory processes and that alterations in this lncRNA may be involved in cancer development and progression.

### 2.7. The Association of VWA8-AS1 with the Tumor Microenvironment (TME)

**Figure 8:**
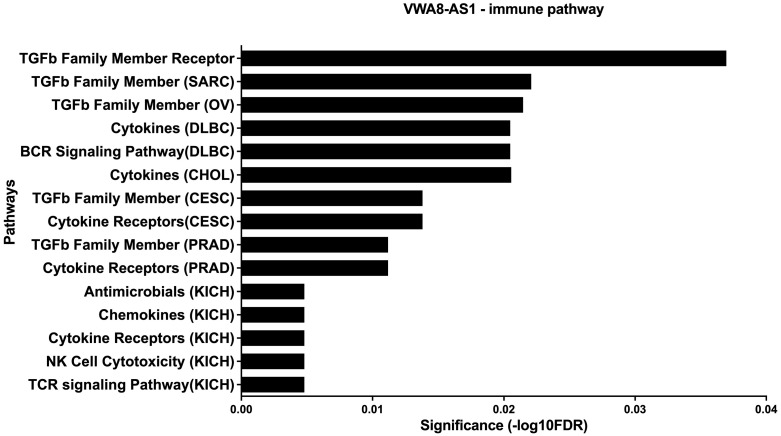
Immune-related pathways of *VWA8-AS1*.

Our findings revealed significant upregulation of *VWA8-AS1* in EBV-infected adjacent tissues, suggesting that EBV-induced *VWA8-AS1* may also play a role in the tumor microenvironment (TME). Data from ImmReg indicated an association between *VWA8-AS1* and immune cell infiltration in cancer. For example, the expression of *VWA8-AS1* was associated with dendritic cells, macrophages, CD8+ T cells, and neutrophils. Moreover, several immune pathways were associated with *VWA8-AS1* (Figure 8). Therefore, *VWA8-AS1* may also play a role in facilitating cancer progression in the TME.

### 2.8. Overexpression of VWA8-AS1 Did Not Affect Oral Cancer Cell Proliferation

**Figure 9:**
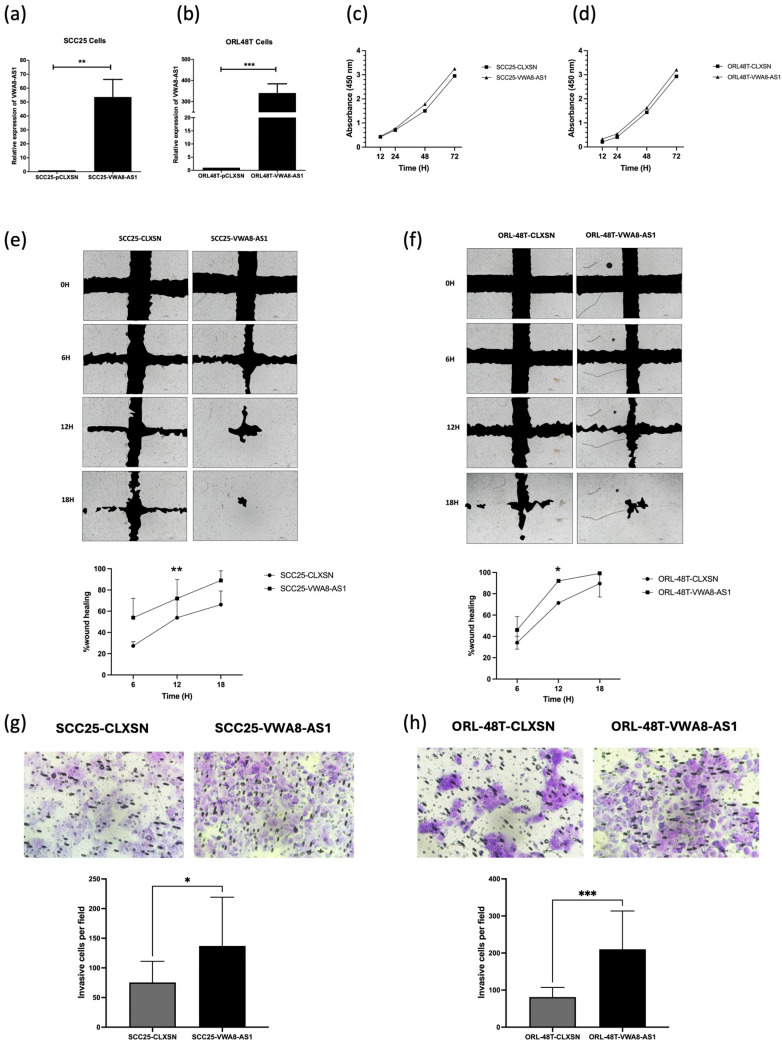
Biological consequence study. (**a**) Relative expression of *VWA8-AS1* in SCC25 cells stably overexpressing *VWA8-AS1* and (**b**) ORL-48T cells by qRT-PCR. (**c**) The proliferation of SCC25 cells overexpressing *VWA8-AS1* and (**d**) *ORL-48T* cells overexpressing *VWA8-AS1* was assessed using the CCK-8 assay. Absorbance was measured at 450 nm at 12, 24, 48, and 72 h after incubation and compared to their respective control cells. The results showed that the overexpression of *VWA8-AS1* did not affect the proliferation of oral cancer cells. (**e**) To investigate the effects of *VWA8-AS1* on the migration of SCC25 cells, and (**f**) ORL-48T cells overexpressing *VWA8-AS1*, a wound healing assay was performed. The extent of wound closure was analyzed at 0, 6, 12, 18, and 24 h and expressed as a percentage relative to the initial wound width; scale bar: 100 μm. Overexpression of *VWA8-AS1* significantly increased the migration of both SCC25 and ORL-48T cells at the 12-h wound closure compared to their respective control counterparts. (**g**) The invasion capability of SCC25 cells and (**h**) ORL-48T cells overexpressing *VWA8-AS1* was analyzed using a Transwell assay, wherein the invaded cells were counted across five fields; Scale bar: 50 μm. Overexpression of *VWA8-AS1* significantly enhanced the invasion of both SCC25 and ORL-48T cells compared to their respective controls. The normality test of data was analyzed using the Shapiro–Wilk test (*p* > 0.05 in all groups), and the data were tested using *t*-tests. (* *p* < 0.05; ** *p* < 0.01; *** *p* < 0.001).

To investigate the biological effects of *VWA8-AS1* on oral cancer cell carcinogenesis, SCC25 and ORL-48T cell lines were employed to establish the stably overexpressed *VWA8-AS1* oral cancer cells (SCC25-*VWA8-AS1* and ORL-48T-*VWA8-AS1*) and their corresponding control cells (SCC25-CLXSN and ORL-48T-CLXSN). As expected, *VWA8-AS1* expression was significantly upregulated in SCC25 and ORL-48T cells transduced with a retrovirus carrying *VWA8-AS1* compared with the control cells (*p* = 0.0058 and *p* = 0.0002, respectively) (Figure 9a,b). These findings suggested that we successfully established stable *VWA8-AS1*-overexpressing oral cancer cells. Our investigation into the proliferation of SCC25-*VWA8-AS1* cells compared to SCC25-CLXSN cells, and in ORL-48T-*VWA8-AS1* cells compared to ORL-48T-CLXSN cells, was performed using a CCK-8 assay. The results revealed no significant change in cell proliferation in either SCC25 or ORL-48T cells overexpressing *VWA8-AS1* compared with that in SCC25 and ORL-48T cells with the vector control (Figure 9c,d). These results indicate that the overexpression of *VWA8-AS1* did not induce oral cancer cell proliferation, suggesting that *VWA8-AS1* may not be involved in this process.

### 2.9. Overexpression of VWA8-AS1 Enhanced the Migration and Invasion of Oral Cancer Cells

Cell migration was evaluated in SCC25-*VWA8-AS1* cells compared to SCC25-CLXSN cells, and in ORL-48T-*VWA8-AS1* cells compared to ORL-48T-CLXSN cells, using a wound healing assay. The results revealed that *VWA8-AS1* overexpression significantly increased the migration of both SCC25-*VWA8-AS1* and ORL-48T-*VWA8-AS1* cells 12 h after incubation, in comparison to their respective control counterparts (*p* = 0.0094 and *p* = 0.0133, respectively) (Figure 9e,f). Moreover, a study of cell invasion was undertaken via a Transwell assay in SCC25-*VWA8-AS1* cells compared to SCC25-CLXSN cells, and in ORL-48T-*VWA8-AS1* cells compared to ORL-48T-CLXSN cells. The results showed that the overexpression of *VWA8-AS1* in oral cancer cells induced cell invasion in both SCC25-*VWA8-AS1* and ORL-48T-*VWA8-AS1* compared to their respective control counterparts (*p* = 0.0403 and *p* = 0.0006, respectively) (Figure 9g,h). These findings suggest that the overexpression of *VWA8-AS1* enhances the migration and invasion of oral cancer cells, thereby promoting tumor progression.

## 3. Discussion

EBV infection is a risk factor for OSCC; however, the underlying mechanisms by which EBV affects OSCC development remain controversial. Recently, lncRNAs associated with cancer have emerged as a research subject in several fields. Interestingly, viruses can alter the expression of lncRNAs as part of their survival and transmission strategies [22,23]. Previous research has also demonstrated that EBV infection can modulate the expression of various lncRNAs, such as *SNHG8* in gastric cancer [37,38] and *MALAT1*, *AFAP1-AS1*, and *AL359062* in nasopharyngeal carcinoma [39]. Therefore, we hypothesized that EBV infection may contribute to the development and progression of OSCC via the modulation of cellular lncRNAs.

In this study, lncRNAs associated with the development of oral cancer in patients with OSCC and EBV infection were identified using RNA-seq datasets of patients with OSCC from a public database and microarray data from EBV-negative and EBV-positive cultured SCC25 cells. We identified several lncRNAs associated with OSCC and EBV infections. Bioinformatic analysis showed that they are associated with cancer progression and development. Consistent with the findings of previous studies, the upregulation of several lncRNAs induces the progression of various cancers by increasing cancer cell proliferation and metastasis. Therefore, the aberrant expression of lncRNAs may play an important role in carcinogenesis. The overexpression of the lncRNA *CASC15* has been reported in several studies and shown to play an oncogenic role by regulating the epithelial-mesenchymal transition (EMT) and enhancing gastric cancer cell proliferation; moreover, *CASC15* is associated with poor patient prognosis [40] and regulates the progression of tongue squamous cell carcinoma by competing with miR-33a-5p [41]. Consequently, *CASC15* can be utilized as a prognostic biomarker and therapeutic target. Regarding renal cell cancer, *LINC00944* contributes to its development by enhancing cancer cell proliferation and migration [42]. However, few studies have been conducted on *LINC00348*, which could be related to bronchopulmonary dysplasia [43] and cutaneous squamous cell carcinoma [44]. *INHBA-AS1* is upregulated in gastric cancer and may be useful in combination with *AK001058*, *MIR4435-2HG*, and *CEBPA-AS1* as diagnostic and prognostic biomarkers [45]. A functional study of *INHBA-AS1* via the lncRNA–miRNA–mRNA axis revealed that this lncRNA promotes cell proliferation in colorectal cancer [46]. This study suggests that EBV may regulate or alter lncRNA expression in carcinogenesis.

Further, the present study analyzed the differential expression of lncRNAs, focusing on those overexpressed in OSCC tissues and associated with EBV infection, including the lncRNA *VWA8-AS1*, which exhibited a high fold change in expression in EBV-positive cells compared to EBV-negative cells. This lncRNA, which has not been previously studied in relation to cancer, was chosen for further investigation to determine its dysregulation in oral cancer cell lines and tissues using qRT-PCR and to study its role in oral carcinogenesis in oral cancer cells. We examined whether EBV infection modulated its expression using cell lines with and without EBV infection. As expected, EBV induced the overexpression of *VWA8-AS1*. Furthermore, we analyzed the expression of *VWA8-AS1* in tumors and adjacent normal tissues. We found that *VWA8-AS1* was significantly overexpressed in EBV-positive tumor tissues, suggesting that EBV regulates the expression of *VWA8-AS1*, which is related to OSCC oncogenesis. Interestingly, the expression of *VWA8-AS1* seemed to be increased in both EBV-infected tumor tissues and infected normal tissues to levels significantly greater than those in EBV-negative tumor tissues. This could be due to EBV infection in the immune cells residing in the microenvironment [47], which also alters the expression of *VWA8-AS1* in the transfected cells. Additionally, data from ImmReg revealed an association between *VWA8-AS1* and the infiltration of several immune cells, such as dendritic cells, macrophages, CD8+ T cells, and neutrophils, in cancer. Therefore, the overexpression of EBV-induced *VWA8-AS1* may not only affect tumor cells but also influence the effects of immune cells in the TME on OSCC development and progression. However, our statistical analysis had relatively low power, and larger sample sizes are needed to draw robust conclusions. These findings suggest that the dysregulation of *VWA8-AS1* induced by EBV in normal adjacent and tumor tissues plays a role in OSCC development and progression and might be initiated in the TME, necessitating molecular functional studies to identify therapeutic targets in EBV-associated OSCC.

Therefore, we focused on a specific lncRNA called *VWA8-AS1* or VWA8 antisense RNA 1 (head-to-head), which is a lncRNA with a length of 2091 nucleotides. The role of *VWA8-AS1* is little-studied since it is still novel; however, it has been linked to various diseases, including atrophic gastritis and gastric cancer [48], thyroid cancer [49], and central nervous system disease [50], by transcriptomic studies. Furthermore, *VWA8-AS1* has been found in exosomes released from gastric cancer cells [51]. The downregulation of *VWA8-AS1* has been observed in T helper cells associated with the immune response in women with polycystic ovary syndrome [52], suggesting a potential role in disease development and progression. Data retrieved from the ImmReg database indicated that *VWA8-AS1* was differentially expressed and related to kidney chromophobe, kidney renal clear-cell carcinoma, lung adenocarcinoma, and thyroid carcinoma [53,54]. However, its role in oral cancer remains unknown.

The functions of lncRNAs are diverse as they can bind to DNA, RNA, and proteins, which leads to varying regulatory effects on various cellular processes. Several functions of lncRNAs have been identified. First, lncRNAs can participate in chromatin modification and remodeling by serving as scaffolds to recruit proteins that modify histones. Second, they can function as transcriptional activators or repressors by interacting with RNA polymerase I or other transcription factors, thus controlling gene transcription. Third, they can act as guides, directing proteins to specific genomic regions to influence gene expression. Fourth, lncRNAs can interact with mRNAs, which can impact their stability, splicing, and translation. Lastly, they can function as miRNA sponges, binding to miRNAs that would typically target specific mRNAs for degradation. Finally, lncRNAs are involved in a range of cellular processes beyond gene expression control [55].

Associations between *VWA8-AS1*, miRNAs, and mRNAs were predicted based on the potential role of lncRNAs in binding to several types of molecules to regulate cellular processes. Functional enrichment analysis was performed to explore the molecular mechanisms of EBV-associated *VWA8-AS1* in OSCC. In our study, we investigated the potential mechanisms by which *VWA8-AS1* is involved in carcinogenesis and cancer progression by predicting its target. We found that the expression levels of several miRNAs were lower in OSCC tissues than in adjacent normal tissues, and some of these miRNAs were predicted to be targeted by *VWA8-AS1*. Specifically, hsa-miR-140-5p, hsa-miR-1-3p, hsa-miR-125b-5p, hsa-miR-127-5p, and hsa-miR-410-3p are well-known tumor suppressor miRNAs. For example, hsa-miR-140-5p directly targets *FEN1* and *YES1* in cervical and gastric cancer, respectively, thereby inhibiting cancer cell proliferation, migration, and invasion [30,33]. The downregulation of hsa-miR-1-3p has been linked to the development and progression of liver and lung cancer. It has been shown to reduce hepatocellular carcinoma cell proliferation by targeting *SOX9* and to suppress lung adenocarcinoma cell tumorigenesis in vivo by binding to *PRC1* [28,32]. Hsa-miR-125b-5p regulates cancer cell growth and proliferation by suppressing the TRAF6/MAPK/NF-κB pathway in osteoarthritic chondrocytes and targeting *MMP1* in breast cancer [29,56]. Hsa-miR-127-5p targets *BCL6* and disrupts NF-κB signaling in breast cancer and hepatocellular carcinoma, inhibiting cancer development [57,58]. Hsa-miR-410-3p upregulation suppresses proliferation, invasion, and migration and promotes apoptosis in rhabdomyosarcoma cells [59]. Additionally, hsa-miR-23b-3p and hsa-miR-136-3p have been shown to exhibit both tumor suppressor and oncogenic properties. For example, hsa-miR-23b-3p has been found to regulate *c-Met* in cervical cancer to inhibit cancer growth [31]. On the other hand, hsa-miR-136-3p promotes the tumorigenesis of glioma by binding to *KLF7* [60]. In contrast, hsa-miR-23b-3p promotes osteosarcoma cell proliferation by binding to *PGC1α* [61], and hsa-miR-136-3p targets *PTEN* to promote gastric cancer metastasis [62]. Moreover, hsa-miR-889-3p has been shown to target genes such as *MNDA* and *KLF9* to promote cell growth and proliferation in osteosarcoma and non-small cell lung cancer, respectively [63,64].

The present study showed that *VWA8-AS1* interacts with several proteins critical for cellular regulation, such as CNOT4, EIF4G2, ELAVL4, HNRNPK, HNRNP, IGF2BP1, IGF2BP2, KHDRBS3, SNRNP70, RC3H1, SRSF1, SRSF9, and FOXA1, which are overexpressed in various cancers and involved in cell proliferation and tumorigenesis. Additionally, *VWA8-AS1* targets TP53, RELA, and MYC, which are crucial for tumor suppression, NF-κB signaling, and oncogenesis, respectively. *VWA8-AS1* also contains binding motifs for EBV and Kaposi’s sarcoma herpesvirus proteins, suggesting that interactions with these viral proteins might regulate its regulation. However, these targets were predicted based on their potential to form interactions; therefore, in vitro studies of the interaction between lncRNA and its target must be conducted to confirm the interaction.

This study investigated the biological effects of overexpressing *VWA8-AS1* in oral cancer cells. The findings revealed that enhancing *VWA8-AS1* in SCC25 and ORL-48T cells increased cancer cell migration and invasion without affecting cell growth. This indicates that *VWA8-AS1* might support the progression of OSCC by controlling the activity of genes linked to spreading, such as *Twist*, *Snail*, *ZEB1*, *ZEB2*, *MMPs*, and integrins, independently of the cell cycle. These alterations can influence the structural and adhesion characteristics of cells, facilitating movement without influencing cell division. Moreover, *VWA8-AS1* might aid cell migration and infiltration by engaging the PI3K-AKT pathway and activating downstream agents like Rac1 and RhoA, which regulate the cell’s structural framework [65]. The MAPK pathway, particularly ERK1/2, may encourage the production of MMPs and impact the organization of the cell’s structure [66,67]. Dysregulating of the Hippo pathway could lead to abnormal expression of YAP/TAZ expression, resulting in the dysregulation of genes associated with the EMT and interactions with the cell matrix [68]. Additionally, it could regulate TGF-β, which can prompt EMT and facilitate cell migration and infiltration by activating various downstream signaling pathways [69].

In summary, this study sheds light on *VWA8-AS1*, whose expression was found to be altered by EBV, potentially linked to the development of OSCC. We confirmed the biological function of *VWA8-AS1*, showing that it did not affect oral cancer cell proliferation but significantly enhanced cell migration and invasion. This could indicate a potential role of *VWA8-AS1* in enhancing tumor progression and warrants its consideration as a therapeutic target. Dysregulation of *VWA8-AS1* induced by EBV in normal adjacent and tumor tissues plays a role in OSCC development and may initiate cancer progression through the TME. This study also revealed potential interactions and possible associations among lncRNAs, miRNAs, and their targets in OSCC, providing valuable information to guide studies on the mechanisms of *VWA8-AS1*’s function in OSCC carcinogenesis. Molecular functional studies will be necessary to gain in-depth knowledge, which could help identify therapeutic targets in EBV-associated OSCC.

## 4. Materials and Methods

### 4.1. Cell Lines

The human tongue squamous cell carcinoma SCC25, poorly differentiated SCC cells, and human skin squamous cell carcinoma HSC1, well-differentiated SCC cells, cell lines were provided by Prof. Hironori Yoshiyama of Shimane University, Japan. Our colleagues previously established the EBV-positive cell lines SCC25-EBV and HSC1-EBV [27]. The human gingival squamous cell carcinoma cell lines, ORL-48T, were kindly provided by Prof. Sok Ching Cheong of the Cancer Research Initiatives Foundation, Sime Darby Medical Centre Jaya, Malaysia. The human epithelial cervical cancer HeLa cell lines and the human embryonic kidney HEK293T cell lines were kindly provided by Prof. Tohru Kiyono of the National Cancer Center Research Institute, Japan. All cell lines were cultured in Dulbecco’s Modified Eagle Medium/F12 (DMEM/F12) (Cat#12500062) (Gibco, Grand Island, NY, USA) supplemented with 10% fetal bovine serum (FBS) (Cat#6140079) (Gibco, Grand Island, NY, USA) and 1% penicillin-streptomycin (Cat#15140122) (Gibco, Grand Island, NY, USA) at 37 °C in a humidified incubator with 5% CO_2_.

### 4.2. Patient Specimens

Formalin-fixed paraffin-embedded OSCC tissue samples obtained at Srinagarind Hospital, Khon Kaen, were retrieved from materials archived at the Department of Pathology, Faculty of Medicine, Khon Kaen University, Thailand, with approval from the Khon Kaen University Ethics Committee (HE631509). EBV infection in the samples was determined using real-time PCR by detecting BALF5 [70], EBNA1 [71], and LMP1 DNA [27] as markers.

### 4.3. RNA-Seq Analysis for the Prediction and Differential Expression of lncRNA in OSCC

RNA-seq datasets for OSCC were retrieved from the European Nucleotide Archive repository, a publicly available online database, under the accession code PRJEB7455 (https://www.ebi.ac.uk/ena/browser/view/PRJEB7455, accessed on 28 February 2021) [72]. The samples were categorized into OSCC tissues and matched normal adjacent tissues from 19 patients (Table S1). RNA-seq analysis for lncRNA prediction was performed using Galaxy (https://usegalaxy.org/, accessed on 28 February 2021) [73], following Iowa State University’s Genome Informatics Facility workflow (https://gif.biotech.iastate.edu, accessed on 15 February 2021). Firstly, the sequence quality checks were conducted with FASTQC (Galaxy Version 0.72+ galaxy1), and adaptors were trimmed using Trimmomatic (Galaxy Version 0.36.5). Next, the trimmed sequence data were mapped to the hg38 reference genome using HISAT2 (Galaxy Version 2.2.1+ galaxy0). Then, the data generated from HISAT2 were subjected to FeatureCounts (Galaxy Version 1.6.3+ galaxy2) to quantify reads per transcript, and LNCipedia 5.2 hg38 annotation was used as a reference [74]. The differentially expressed genes (DEGs) were analyzed using DESeq2 (DEApps) version 3.9 [75]. A volcano plot was generated using SRplot (https://www.bioinformatics.com.cn/en, accessed on 9 March 2024) [76] with a *p*-value < 0.001 and a fold-change cutoff of 2 to select the DEGs for further analysis.

### 4.4. Microarray Data and Microarray Analysis

Microarray data analyses were previously conducted by our colleagues from established SCC25 cells and EBV-positive SCC25 cells [26]. Briefly, RNA was extracted with ISOGEN reagent (Nippon Gene, Tokyo, Japan) per the manufacturer’s instructions, quantified, and quality checked with a Qubit 2.0 fluorometer (Invitrogen, Carlsbad, CA, USA) and a 2100 Bioanalyzer (Agilent, Santa Clara, CA, USA). The Agilent G4900DA SureScan Microarray Scanner System (Agilent, Santa Clara, CA, USA) was used following the manufacturer’s protocol. The microarray detected mRNAs and more than 14,000 lncRNAs, including those in databases such as the BROAD Institute lincRNA, Lncipedia, and Ensembl. The microarray data analysis was conducted by DNA Chip Research Inc. (Tokyo, Japan). Genes with a fold change above 1.5 were chosen for further analysis, and a heatmap was created with the ggplot2 R package version 3.4.4 [77].

### 4.5. qRT-PCR

Total RNA was extracted from cell lines and tissues using TRIzol reagent (Cat#15596018) (Invitrogen, Carlsbad, CA, USA). cDNA was synthesized from 1 µg of RNA using a RevertAid First Strand cDNA Synthesis Kit (Cat#K1622) (Invitrogen, Carlsbad, CA, USA) and random hexamers (Cat#N8080127) (Invitrogen, Carlsbad, CA, USA). qRT-PCR was performed using SsoAdvancedTM SYBR^®^ Green Supermix (Cat#725271) (Bio-Rad, Hercules, CA, USA) and an Applied Biosystems QuantStudio 6 Flex Real-Time PCR System (Applied Biosystems, Foster City, CA, USA), with Glyceraldehyde 3-phosphate dehydrogenase (*GAPDH*) as an internal control. The relative mRNA expression levels were quantified using the 2^−ΔΔCT^ method [78]. The primers used in this study were designed using the Primer-Blast tool (https://www.ncbi.nlm.nih.gov/tools/primer-blast/index.cgi, accessed on 24 March 2021) [79]. The amplification cycle included the first step for 5 min at 95 °C for initial denaturation, followed by 45 cycles of denaturation cycle for 10 s at 95 °C and annealing cycle for 30 s at 64 °C for BALF5, 55 °C for EBNA1, and 60 °C for LMP1, *VWA8-AS1*, and *GAPDH*. The primer sequences are shown in Table 3

### 4.6. Establishment of VWA8-AS1-Overexpressing SCC25 and ORL-48T Cells

The plasmid pUC57, containing *VWA8-AS1* (NR_039974.1), was synthesized (Bio Basic Inc., Markham, ON, Canada), cloned, and inserted into the pDORN221 vector via recombinational cloning with Gateway BP (Cat#11789013) and LR clonases (Cat#11791020) (Invitrogen, Carlsbad, CA, USA). The plasmid was subsequently transferred into the pCLXSN vector from Prof. Tohru Kiyono. To create a retroviral expression vector, the clone was co-transfected with the GAG-Pol and VSV-G plasmids into HEK293T cells using jetOPTIMUS^®^ transfection reagent (Cat#101000051) (Polyplus-transfection SA, Illkirch, France). Seventy-two hours post-transfection, the medium containing the retrovirus was harvested and stored at −80 °C. The viral titer was quantified using a colony-forming unit assay with HeLa cells. Viral solutions were then added to SCC25 and ORL-48T cell lines, which were subsequently cultured with polybrene. The infection occurred in humidified air with 5% CO_2_ at 37 °C. After 48 h, a medium supplemented with G418 (Cat#4727878001) (Roche Diagnostics Deutschland GmbH, Mannheim, Germany) (400 µg/mL for SCC25 cells and 200 µg/mL for ORL-48T cells) was used to select resistant clones for 10–14 days.

### 4.7. Differential Expression of miRNAs in OSCC

miRNA transcriptome expression data of head and HNSCC patients [80] were obtained from The Cancer Genome Atlas (TCGA) (https://tcga-data.nci.nih.gov/tcga/tcgaDownload.jsp, accessed on 16 October 2021). The retrieved miRNA transcriptome profiling samples were categorized into two distinct groups: 16 cases representing normal tissue and 16 cases representing cancerous tissue (Supplementary File S1). The miRNA transcriptome data of the cancerous tissue group was selected based on tissues related to oral cancer, including the lip, floor of the mouth, tongue, gum, buccal mucosa, and hard palate. The miRNA read count data were analyzed for differential expression using DESeq2 (DEApps) version 3.9 (https://yanli.shinyapps.io/DEApp, accessed on 16 October 2021) [75], comparing cancerous and adjacent normal tissue groups. A volcano plot was constructed using SRplot (https://www.bioinformatics.com.cn/en, accessed on 9 March 2024) [76] with a *p*-value < 0.05 and a fold-change cutoff of 1.5 to select the DEGs for further analysis.

### 4.8. VWA8-AS1 Binding Target Prediction Tools

The interaction of the lncRNA *VWA8-AS1* was analyzed via DIANA LncRBase version 2.0 (https://dianalab.e-ce.uth.gr/html/diana/web/index.php?r=lncbasev2, accessed on 24 February 2022) [81]. Predicted scores > 0.7 were selected for further analysis. The selected miRNAs from prediction which showed low expression levels in OSCC from DEGs of transcriptomic data analysis were selected for mRNA target prediction via MiRDB (https://mirdb.org/, accessed on 9 March 2024) [82,83] with predicted score ≥ 50, mirDIP Version 5.3.0.2, Database version 5.2.3.1 (https://ophid.utoronto.ca/mirDIP/, accessed on 9 March 2024) [84,85] with high to very high predicted score, miRTarBase (https://mirtarbase.cuhk.edu.cn/~miRTarBase/miRTarBase_2019/php/index.php, accessed on 9 March 2024) [86] with at least one binding site, and TargetScan version 8.0 (https://www.targetscan.org/vert_80/, accessed on 9 March 2024) [87] with at least one binding site. These mRNAs were compared with the significantly upregulated mRNAs from the microarray data of EBV-positive SCC25 cells. Potential binding targets of *VWA8-AS1* were predicted using RBPmap version 1.2 (https://rbpmap.technion.ac.il, accessed on 9 March 2024) [88], with z-score ≥ 2 and *p*-value < 0.05, and RNAINTER version 4.0 (http://www.rnainter.org, accessed on 9 March 2024) [89]. The interaction network was constructed using Cytoscape software version 3.7.2 [90].

### 4.9. GO and KEGG Pathway Analysis

GO terms were used to annotate and classify the gene functions. A list of targets associated with lncRNA was used as the subject for annotation to identify the molecular functions represented in the gene profile. KEGG analysis was used for pathway analysis in Panther (http://pantherdb.org/webservices/go/overrep.jsp, accessed on 14 March 2024) [91] to identify the molecular functions represented in the gene profile.

### 4.10. Immune Cell Pathways Analysis

The immune-related pathways in cancers were analyzed in ImmReg (http://bio-bigdata.hrbmu.edu.cn/ImmReg/, accessed on 14 March 2024) [53,54] to identify the enrichment of immune cell pathways and infiltrating immune cells in cancers for *VWA8-AS1*.

### 4.11. Cell Counting Kit-8 (CCK-8) Assay

Cell proliferation was assessed with a CCK-8 assay (Cat#CK04-05) (CCK-8, DOJINDO, Kumamoto, Japan). The cells were dispensed into 96-well plates (100 μL/well, 3000 cells/well) and incubated at 37 °C for 12, 24, 48, and 72 h. Subsequently, 10 µL/well of CCK-8 solution was added and incubated for 1–4 h. The absorbance was measured at 450 nm using a Varioskan LUX Multimode Microplate Reader (Thermo Scientific, Waltham, MA, USA).

### 4.12. Wound Healing Assay

The cells were seeded in 24-well plates at 2 × 10^5^ cells/well in DMEM/F12 medium with 10% FBS and incubated at 37 °C under 5% CO_2_ until 100% confluence. A linear wound was created using sterile 200 µL pipette tips, and cells were maintained in DMEM/F12 supplemented with 1% FBS. The wound closure of five regions per created wound was observed at 0, 6, 12, 18, and 24 h using a Nikon inverted fluorescence microscope ECLIPSE-Ti2-U (Nikon Corporation, Tokyo, Japan) and NIS-Elements Advanced Research Imaging Software version 3.0 (Nikon Corporation, Tokyo, Japan). The extent of wound closure was analyzed using ImageJ version 1.53k [92] and converted to a percentage relative to the initial wound width.

### 4.13. Transwell Assay

A Transwell^®^ insert with a 0.8 µm NEST^®^ polycarbonate membrane was coated with 50 uL of 1mg/mL Matrigel Matrix (Cat#354234) (Corning, Bedford, MA, USA) and seeded with 1 × 10^5^ cells/well in a serum-free medium. Following 24 h of incubation at 5% CO_2_, noninvasive cells were removed from the upper surface, while cells that invaded the matrix gel and attached to the lower surface were fixed with 70% ethanol, stained with 0.2% crystal violet, photographed, and counted in five fields. A Nikon inverted fluorescence microscope ECLIPSE-Ti2-U (Nikon Corporation, Tokyo, Japan) and NIS-Elements Advanced Research Imaging Software version 3.0 (Nikon Corporation, Tokyo, Japan) were used to capture images.

### 4.14. Statistical Analysis

GraphPad Prism version 10.1.1 (GraphPad Software Inc., San Diego, CA, USA) was used for data analysis. The Shapiro–Wilk test was used for analysis of data normality distribution. Mann–Whitney U tests and *t*-tests were used to analyze and to test whether there was a difference between two independent groups. All experiments were repeated three times. Statistical significance was set at *p* < 0.05.

## 5. Conclusions

In conclusion, this investigation elucidated several key findings regarding *VWA8-AS1* in OSCC. Firstly, *VWA8-AS1* expression was observed to be upregulated in response to EBV infection. Secondly, dysregulation of EBV-induced *VWA8-AS1* in both tumor and adjacent normal tissues contributes to OSCC formation and may accommodate cancer advancement via the tumor microenvironment, suggesting an association with OSCC progression. Thirdly, overexpression of *VWA8-AS1* enhanced cancer cell migration and invasion, potentially contributing to metastasis. Fourthly, interactions with regulatory proteins and tumor-associated genes suggest the involvement of a complex molecular network. Lastly, predicted interactions with miRNAs indicate an additional mechanism for cancer progression. This study highlights the potential importance of *VWA8-AS1* in the development and progression of OSCC, suggesting new pathways for future research into comprehensive molecular investigations related to oral cancer biology and possibly guiding novel diagnostic or therapeutic strategies.

## Figures and Tables

**Table 1:** Thirty upregulated or downregulated lncRNAs in EBV-positive oral cancer cells compared with EBV-negative SCC25 cells, and statistically significant differences in the RNA-seq data of OSCC tissues.

SCC25-EBV vs. SCC25			
LncRNA	Log2FC	LncRNA	Log2FC
*lnc-PRR16-1*	5.43	*ENO1-AS1*	−4.64
*VWA8-AS1*	4.01	*LINC01124*	−4.08
*lnc-FADD-1*	4.00	*lnc-PRL-7*	−3.96
*LINC00944*	3.76	*lnc-CTNNA2-2*	−3.53
*lnc-REEP5-1*	3.74	*lnc-NCOA3-4*	−3.50
*lnc-SNX18-1*	3.58	*lnc-SLC7A7-1*	−3.40
*EXTL3-AS1*	3.53	*HOXA-AS2*	−3.34
*lnc-ABCD3-2*	3.53	*LURAP1L-AS1*	−3.32
*lnc-ARFGEF2-4*	3.51	*LINC00629*	−3.26
*SPACA6P-AS*	3.35	*lnc-COPZ2-1*	−3.22
*LINC00348*	3.34	*HOTAIR*	−3.20
*LINC00499*	3.29	*lnc-IL4R-2*	−3.18
*lnc-SEPT9-3*	3.24	*LINC00518*	−3.12
*INHBA-AS1*	3.20	*lnc-PEX2-3*	−3.06
*lnc-RTL1-6*	3.11	*CDKN2B-AS1*	−3.00
*LINC01120*	3.07	*CRTC3-AS1*	−2.83
*JRKL-AS1*	3.07	*lnc-RELT-1*	−2.81
*lnc-RPP30-2*	2.97	*lnc-C10orf90-2*	−2.72
*lnc-HNF1A-1*	2.95	*MNX1-AS1*	−2.70
*lnc-GNG8-1*	2.95	*TGFB2-AS1*	−2.65
*LINC00456*	2.92	*LINC01569*	−2.64
*lnc-CEBPB-6*	2.91	*FAM87A*	−2.64
*lnc-TTLL2-1*	2.87	*ERICH6-AS1*	−2.61
*lnc-TCL1B-2*	2.82	*CASC11*	−2.58
*lnc-PIK3CG-3*	2.81	*SNAI3-AS1*	−2.56
*CASC15*	2.77	*lnc-REG3G-3*	−2.52
*LINC01488*	2.53	*lnc-CHAF1B-3*	−2.50
*lnc-GNA12-2*	2.44	*WWC2-AS2*	−2.47
*lnc-SECTM1-1*	2.27	*lnc-NR1I2-1*	−2.46
*lnc-NVL-3*	2.20	*ZBED3-AS1*	−2.42

SCC25: EBV-negative tongue squamous cell carcinoma; SCC25-EBV: EBV-positive tongue squamous cell carcinoma.

**Table 2:** Interacting *VWA8-AS1*, miRNA, and mRNA targets.

miRNA	mRNA Targets
hsa-miR-1-3p	*ZNF24 C2orf69 NAMPT FNDC3B SMIM14 HSP90B1 ARF3 FBXO33 CAPRIN1 SOX9 MMD RRBP1 GCH1 MON2 TRIM2 AKAP12 CHST11 HIPK3 EPB41L2 FGD4 RIMS4 SH3BGRL3 ARF4*
hsa-miR-23b-3p	*TNFAIP6 AMBRA1 TXNRD1 BNIP3L ZNF292 CAPRIN1 SUCO BORA RAB8B C2orf69 ROBO2 RALYL IL6R NFIB UBE2D3 POU2F1 APAF1 CNOT6L CCNG1 UBE2D1 CLDN12 ZBTB34 WBP2 HEXIM1 SEC24A PPM1A CELF1 ZNF267 PDIA6 MYM2 DCP2 RBPMS2 TAB2 TNFAIP3 NUFIP2 ETNK1 RNF38*
hsa-miR-125b-5p	*SLC26A6 LBH SERTAD3 NEU1 ABCC5 PPP2R5C NFIB CNNM1 RAPGEF5 ANPEP FNDC3B SYVN1 LIFR PRRC1 ELOVL6 CEP85 CRB2 LACTB IKZF4 ERBB3 ARID3A STOX2 HOXD1 YOD1 ANKRD33B BTG2 TNFAIP3 BMF MEGF9 TET2 ARID3B IL6R RPS6KA1 CGN KCNS3 BAK1 TP53INP1 ZNF385A PRDM1*
hsa-miR-127-5p	*EPAS1 DDX3Y ATP2B4 MMP13 MTSS1 PCDHA5 HIPK3 CNOT2 NFIB EREG UBFD1 STK17B YOD1 SNX10*
hsa-miR-136-3p	*VASN ZNF292 PHF21B NFIB TAB2*
hsa-miR-140-5p	*TTYH3 EGR2 SHROOM3 ZBTB10 PPTC7 SMURF1 BAZ2B MTSS1 HSPA4L KLF6 CALU SLC30A5 ADCY6 DCUN1D3 GALNT16 PRDM1 YOD1 MMD*
hsa-miR-410-3p	*ADM ARHGEF40 FBXO33 RAB8B BAZ2B RORA TRAPPC3 ATP8B2 RGMB TTYH3 GTF2B BTG3 KLF6*
hsa-miR-889-3p	*TTYH3 SHROOM3 MTSS1 KLF6 SMURF1 HSPA4L ZBTB10 BAZ2B SLC30A5 DCUN1D3 EPB41L2 GALNT16 KLF9 EGR2 ZNF800 PPTC7 CALU ADCY6 PRDM1 MMD YOD1*

**Table 3:** Primer sequences.

Gene Name	Forward (5′–3′)	Reverse (5′–3′)
BALF5	CGAGTCATCTACGGGGACACGGA	AGCACCCCCACATATCTCTTCTT
EBNA1	CCACAATGTCGTCTTACACC	ATAACAGACAATGGACTCCCT
LMP1	TCTCCTTTGGCTCCTCCTGT	TCGGTAGCTTGTTGAGGGTG
*VWA8-AS1*	GCCTGACCCTGTGATGAGTG	TCCTCAAGTTCTTCCGCTGG
*GAPDH*	TCATCAGCAATGCCTCCTGCA	TGGGTAGCAGTGATGGCA

## Data Availability

The RNA-seq datasets used in the current study were retrieved from the European Nucleotide Archive repository under the accession code PRJEB7455 (https://www.ebi.ac.uk/ena/browser/view/PRJEB7455 accessed on 28 February 2021). The microarray datasets used in the current study are available from the corresponding author upon reasonable request. The miRNA transcriptome expression data used in this study were retrieved from The Cancer Genome Atlas (https://tcga-data.nci.nih.gov/tcga/tcgaDownload.jsp accessed on 16 October 2021).

## Supplementary Materials

The following supporting information can be downloaded at: https://www.mdpi.com/article/10.3390/ijms252312565/s1.

## References

[1] Kumar M., Nanavati R., Modi T., Dobariya C. (2016). Oral Cancer: Etiology and Risk Factors: A Review. J. Cancer Res. Ther..

[2] Cruz I., Van Den Brule A.J.C., Steenbergen R.D.M., Snijders P.J.F., Meijer C.J.L.M., Walboomers J.M.M., Snow G.B., Van Der Waal I. (1997). Prevalence of Epstein—Barr Virus in Oral Squamous Cell Carcinomas, Premalignant Lesions and Normal Mucosa—A Study Using the Polymerase Chain Reaction. Oral Oncol..

[3] Jalouli J., Jalouli M.M., Sapkota D., Ibrahim S.O., Larsson P.-A., Sand L. (2012). Human Papilloma Virus, Herpes Simplex Virus and Epstein Barr Virus in Oral Squamous Cell Carcinoma from Eight Different Countries. Anticancer Res..

[4] Acharya S., Ekalaksananan T., Vatanasapt P., Loyha K., Phusingha P., Promthet S., Kongyingyoes B., Pientong C. (2015). Association of Epstein-Barr Virus Infection with Oral Squamous Cell Carcinoma in a Case—Control Study. J. Oral Pathol. Med..

[5] Maymone M.B.C., Greer R.O., Kesecker J., Sahitya P.C., Burdine L.K., Cheng A.-D., Maymone A.C., Vashi N.A. (2019). Premalignant and Malignant Oral Mucosal Lesions: Clinical and Pathological Findings. J. Am. Acad. Dermatol..

[6] Kröplin J., Reppenhagen J.-C. (2024). Best Practices and Future Challenges in the Treatment of Oral Cancer. Innov. Surg. Sci..

[7] Vockerodt M., Yap L., Shannon-Lowe C., Curley H., Wei W., Vrzalikova K., Murray P.G. (2015). The Epstein–Barr Virus and the Pathogenesis of Lymphoma. J. Pathol..

[8] Tsao S., Tsang C.M., To K., Lo K. (2015). The Role of Epstein—Barr Virus in Epithelial Malignancies. J. Pathol..

[9] Gupta K., Metgud R. (2013). Evidences Suggesting Involvement of Viruses in Oral Squamous Cell Carcinoma. Pathol. Res. Int..

[10] Gondivkar S.M., Parikh R.V., Gadbail A.R., Solanke V., Chole R., Mankar M., Balsaraf S. (2012). Involvement of Viral Factors with Head and Neck Cancers. Oral Oncol..

[11] Majumdar B. (2017). Etiologic Association between Epstein–Barr Virus and Oral Squamous Cell Carcinoma: A Brief Evidence-Based Discussion. J. Contemp. Dent. Pract..

[12] Yen C.-Y., Lu M.-C., Tzeng C.-C., Huang J.-Y., Chang H.-W., Chen R.-S., Liu S.-Y., Liu S.-T., Shieh B., Li C. (2009). Detection of EBV Infection and Gene Expression in Oral Cancer from Patients in Taiwan by Microarray Analysis. BioMed Res. Int..

[13] Shimakage M., Horii K., Tempaku A., Kakudo K., Shirasaka T., Sasagawa T. (2002). Association of Epstein-Barr Virus with Oral Cancers. Hum. Pathol..

[14] Kikuchi K., Noguchi Y., De Rivera M.W.G.-N., Hoshino M., Sakashita H., Yamada T., Inoue H., Miyazaki Y., Nozaki T., González-López B.S. (2016). Detection of Epstein-Barr Virus Genome and Latent Infection Gene Expression in Normal Epithelia, Epithelial Dysplasia, and Squamous Cell Carcinoma of the Oral Cavity. Tumor Biol..

[15] Budhy T.I. (2018). Molecular Grading of Oral Squamous Cell Carcinomas Infected with EBV. Asian Pac. J. Cancer Prev..

[16] Meckes D.G. (2011). Mining Epstein-Barr Virus LMP1 Signaling Networks. J. Carcinog. Mutagen..

[17] Queen K.J., Shi M., Zhang F., Cvek U., Scott R.S. (2013). Epstein–Barr Virus-induced Epigenetic Alterations Following Transient Infection. Int. J. Cancer.

[18] Higa M., Kinjo T., Kamiyama K., Chinen K., Iwamasa T., Arasaki A., Sunakawa H. (2003). Epstein–Barr Virus (EBV)-Related Oral Squamous Cell Carcinoma in Okinawa, a Subtropical Island, in Southern Japan—Simultaneously Infected with Human Papillomavirus (HPV). Oral Oncol..

[19] Gibb E.A., Brown C.J., Lam W.L. (2011). The Functional Role of Long Non-Coding RNA in Human Carcinomas. Mol. Cancer.

[20] Fernandes J.C.R., Acuña S.M., Aoki J.I., Floeter-Winter L.M., Muxel S.M. (2019). Long Non-Coding RNAs in the Regulation of Gene Expression: Physiology and Disease. ncRNA.

[21] Irimie A.I., Braicu C., Sonea L., Zimta A.A., Cojocneanu-Petric R., Tonchev K., Mehterov N., Diudea D., Buduru S., Berindan-Neagoe I. (2017). A Looking-Glass of Non-Coding RNAs in Oral Cancer. Int. J. Mol. Sci..

[22] Liu W., Zhang Y., Luo B. (2021). Long Non-Coding RNAs in Gammaherpesvirus Infections: Their Roles in Tumorigenic Mechanisms. Front. Microbiol..

[23] Huang Y., Jiang L., Liu Y., Liu L., Wang J., Shi L. (2022). Long Non-coding RNAs in Virus-related Cancers. Rev. Med. Virol..

[24] Zhang J., Zhang S., Zuo L., Yue W., Li S., Xin S., Liu L., Lu J. (2019). Differential Expression Profiling of lncRNAs Related to Epstein-Barr Virus Infection in the Epithelial Cells. J. Med. Virol..

[25] Zhang J., Li X., Hu J., Cao P., Yan Q., Zhang S., Dang W., Lu J. (2020). Long Noncoding RNAs Involvement in Epstein-Barr Virus Infection and Tumorigenesis. Virol. J..

[26] Heawchaiyaphum C., Pientong C., Yoshiyama H., Iizasa H., Panthong W., Ekalaksananan T. (2021). General Features and Novel Gene Signatures That Identify Epstein-Barr Virus-Associated Epithelial Cancers. Cancers.

[27] Heawchaiyaphum C., Iizasa H., Ekalaksananan T., Burassakarn A., Kiyono T., Kanehiro Y., Yoshiyama H., Pientong C. (2020). Epstein–Barr Virus Infection of Oral Squamous Cells. Microorganisms.

[28] Li T., Wang X., Jing L., Li Y. (2019). MiR-1-3p Inhibits Lung Adenocarcinoma Cell Tumorigenesis via Targeting Protein Regulator of Cytokinesis 1. Front. Oncol..

[29] Wang Y., Wei Y., Fan X., Zhang P., Wang P., Cheng S., Zhang J. (2020). MicroRNA-125b as a Tumor Suppressor by Targeting MMP11 in Breast Cancer. Thorac. Cancer.

[30] Guo Y., Luo S. (2020). miR-140-5p Inhibits Cervical Cancer Cell Phenotypes via Downregulating FEN1 to Halt the Cell Cycle. Mol. Med. Rep..

[31] Campos-Viguri G.E., Peralta-Zaragoza O., Jiménez-Wences H., Longinos-González A.E., Castañón-Sánchez C.A., Ramírez-Carrillo M., Camarillo C.L., Castañeda-Saucedo E., Jiménez-López M.A., Martínez-Carrillo D.N. (2020). MiR-23b-3p Reduces the Proliferation, Migration and Invasion of Cervical Cancer Cell Lines via the Reduction of c-Met Expression. Sci. Rep..

[32] Zhang H., Zhang Z., Gao L., Qiao Z., Yu M., Yu B., Yang T. (2019). miR-1-3p Suppresses Proliferation of Hepatocellular Carcinoma through Targeting SOX9. OTT.

[33] Fang Z., Yin S., Sun R., Zhang S., Fu M., Wu Y., Zhang T., Khaliq J., Li Y. (2017). miR-140-5p Suppresses the Proliferation, Migration and Invasion of Gastric Cancer by Regulating YES1. Mol. Cancer.

[34] Shiah S.-G., Hsiao J.-R., Chang W.-M., Chen Y.-W., Jin Y.-T., Wong T.-Y., Huang J.-S., Tsai S.-T., Hsu Y.-M., Chou S.-T. (2014). Downregulated miR329 and miR410 Promote the Proliferation and Invasion of Oral Squamous Cell Carcinoma by Targeting Wnt-7b. Cancer Res..

[35] Fukumoto I., Koshizuka K., Hanazawa T., Kikkawa N., Matsushita R., Kurozumi A., Kato M., Okato A., Okamoto Y., Seki N. (2016). The Tumor-Suppressive microRNA-23b/27b Cluster Regulates the MET Oncogene in Oral Squamous Cell Carcinoma. Int. J. Oncol..

[36] Henson B.J., Bhattacharjee S., O’Dee D.M., Feingold E., Gollin S.M. (2009). Decreased Expression of miR-125b and miR-100 in Oral Cancer Cells Contributes to Malignancy. Genes Chromosomes Cancer.

[37] Liu J., Yang C., Gu Y., Li C., Zhang H., Zhang W., Wang X., Wu N., Zheng C. (2018). Knockdown of the lncRNA SNHG8 Inhibits Cell Growth in Epstein-Barr Virus-Associated Gastric Carcinoma. Cell. Mol. Biol. Lett..

[38] Huang T., Ji Y., Hu D., Chen B., Zhang H., Li C., Chen G., Luo X., Zheng X., Lin X. (2016). SNHG8 Is Identified as a Key Regulator of Epstein-Barr Virus(EBV)-Associated Gastric Cancer by an Integrative Analysis of lncRNA and mRNA Expression. Oncotarget.

[39] He B., Zeng J., Chao W., Chen X., Huang Y., Deng K., Huang Z., Li J., Dai M., Chen S. (2017). Serum Long Non-Coding RNAs MALAT1, AFAP1-AS1 and AL359062 as Diagnostic and Prognostic Biomarkers for Nasopharyngeal Carcinoma. Oncotarget.

[40] Wu Q., Xiang S., Ma J., Hui P., Wang T., Meng W., Shi M., Wang Y. (2018). Long Non-coding RNA CASC 15 Regulates Gastric Cancer Cell Proliferation, Migration and Epithelial Mesenchymal Transition by Targeting CDKN 1A and ZEB 1. Mol. Oncol..

[41] Zuo Z., Ma L., Gong Z., Xue L., Wang Q. (2018). Long Non-Coding RNA CASC15 Promotes Tongue Squamous Carcinoma Progression through Targeting miR-33a-5p. Environ. Sci. Pollut. Res..

[42] Chen C., Zheng H. (2021). LncRNA LINC00944 Promotes Tumorigenesis but Suppresses Akt Phosphorylation in Renal Cell Carcinoma. Front. Mol. Biosci..

[43] Zhang Z., Chen K., Pan D., Liu T., Hang C., Ying Y., He J., Lv Y., Ma X., Chen Z. (2023). A Predictive Model for Preterm Infants with Bronchopulmonary Dysplasia Based on Ferroptosis-Related lncRNAs. BMC Pulm. Med..

[44] Hu Y., Li R., Chen H., Chen L., Zhou X., Liu L., Ju M., Chen K., Huang D. (2022). Comprehensive Analysis of lncRNA-mRNAs Co-Expression Network Identifies Potential lncRNA Biomarkers in Cutaneous Squamous Cell Carcinoma. BMC Genom..

[45] Ke D., Li H., Zhang Y., An Y., Fu H., Fang X., Zheng X. (2017). The Combination of Circulating Long Noncoding RNAs AK001058, INHBA-AS1, MIR4435-2HG, and CEBPA-AS1 Fragments in Plasma Serve as Diagnostic Markers for Gastric Cancer. Oncotarget.

[46] Lin H., Hong Y.-G., Zhou J.-D., Gao X.-H., Yuan P.-H., Xin C., Huang Z.-P., Zhang W., Hao L.-Q., Hou K.-Z. (2020). LncRNA INHBA-AS1 Promotes Colorectal Cancer Cell Proliferation by Sponging miR-422a to Increase AKT1 Axis. Eur. Rev. Med. Pharmacol. Sci..

[47] Tan G., Visser L., Tan L., Berg A., Diepstra A. (2018). The Microenvironment in Epstein–Barr Virus-Associated Malignancies. Pathogens.

[48] Liu S., Yin H., Zheng S., Chu A., Li Y., Xing C., Yuan Y., Gong Y. (2020). Differentially Expressed mRNAs and Their Long Noncoding RNA Regulatory Network with Helicobacter Pylori-Associated Diseases Including Atrophic Gastritis and Gastric Cancer. BioMed Res. Int..

[49] Zhang Y., Jin T., Shen H., Yan J., Guan M., Jin X. (2019). Identification of Long Non-Coding RNA Expression Profiles and Co-Expression Genes in Thyroid Carcinoma Based on The Cancer Genome Atlas (TCGA) Database. Med. Sci. Monit..

[50] Hicks C., Sitthi-Amorn J., Douglas J., Ramani R., Miele L., Vijayakumar V., Karlson C., Chipeta J., Megason G. (2016). Molecular Analysis of Central Nervous System Disease Spectrum in Childhood Acute Lymphoblastic Leukemia. Clin. Med. Insights Oncol..

[51] Jin G., Zhang J., Cao T., Chen B., Tian Y., Shi Y. (2022). Exosome-Mediated lncRNA SND1-IT1 from Gastric Cancer Cells Enhances Malignant Transformation of Gastric Mucosa Cells via up-Regulating SNAIL1. J. Transl. Med..

[52] Hiam D., Simar D., Laker R., Altıntaş A., Gibson-Helm M., Fletcher E., Moreno-Asso A., Trewin A.J., Barres R., Stepto N.K. (2019). Epigenetic Reprogramming of Immune Cells in Women with PCOS Impact Genes Controlling Reproductive Function. J. Clin. Endocrinol. Metab..

[53] Jiang T., Zhou W., Chang Z., Zou H., Bai J., Sun Q., Pan T., Xu J., Li Y., Li X. (2021). ImmReg: The Regulon Atlas of Immune-Related Pathways across Cancer Types. Nucleic Acids Res..

[54] Li Y., Jiang T., Zhou W., Li J., Li X., Wang Q., Jin X., Yin J., Chen L., Zhang Y. (2020). Pan-Cancer Characterization of Immune-Related lncRNAs Identifies Potential Oncogenic Biomarkers. Nat. Commun..

[55] Statello L., Guo C.-J., Chen L.-L., Huarte M. (2021). Gene Regulation by Long Non-Coding RNAs and Its Biological Functions. Nat. Rev. Mol. Cell Biol..

[56] Rasheed Z., Rasheed N., Abdulmonem W.A., Khan M.I. (2019). MicroRNA-125b-5p Regulates IL-1β Induced Inflammatory Genes via Targeting TRAF6-Mediated MAPKs and NF-κB Signaling in Human Osteoarthritic Chondrocytes. Sci. Rep..

[57] Chen J., Wang M., Guo M., Xie Y., Cong Y.-S. (2013). miR-127 Regulates Cell Proliferation and Senescence by Targeting BCL6. PLoS ONE.

[58] Wu J., Ding J., Yang J., Guo X., Zheng Y. (2018). MicroRNA Roles in the Nuclear Factor Kappa B Signaling Pathway in Cancer. Front. Immunol..

[59] Zhang L., Pang Y., Cui X., Jia W., Cui W., Liu Y., Liu C., Li F. (2019). MicroRNA-410-3p Upregulation Suppresses Proliferation, Invasion and Migration, and Promotes Apoptosis in Rhabdomyosarcoma Cells. Oncol. Lett..

[60] Xu Y. (2020). MicroRNA-136-3p Inhibits Glioma Tumorigenesis in Vitro and in Vivo by Targeting KLF7. World J. Surg. Oncol..

[61] Zhu R., Li X., Ma Y. (2019). miR-23b-3p Suppressing PGC1α Promotes Proliferation through Reprogramming Metabolism in Osteosarcoma. Cell Death Dis..

[62] Chen X., Huang Z., Chen R. (2018). Microrna-136 Promotes Proliferation and Invasion Ingastric Cancer Cells through Pten/Akt/P-Akt Signaling Pathway. Oncol. Lett..

[63] Ge D., Chen H., Zheng S., Zhang B., Ge Y., Yang L., Cao X. (2019). Hsa-miR-889-3p Promotes the Proliferation of Osteosarcoma through Inhibiting Myeloid Cell Nuclear Differentiation Antigen Expression. Biomed. Pharmacother..

[64] Han X., Tang Y., Dai Y., Hu S., Zhou J., Liu X., Zhu J., Wu Y. (2019). MiR-889 Promotes Cell Growth in Human Non-Small Cell Lung Cancer by Regulating KLF9. Gene.

[65] Devreotes P., Horwitz A.R. (2015). Signaling Networks That Regulate Cell Migration. Cold Spring Harb. Perspect. Biol..

[66] Samson S.C., Khan A.M., Mendoza M.C. (2022). ERK Signaling for Cell Migration and Invasion. Front. Mol. Biosci..

[67] Tsai J.H., Yang J. (2013). Epithelial-Mesenchymal Plasticity in Carcinoma Metastasis. Genes Dev..

[68] Zhou T., Li X., Liu J., Hao J. (2023). The Hippo/YAP Signaling Pathway: The Driver of Cancer Metastasis. Cancer Biol. Med..

[69] Yang Y., Ye W.-L., Zhang R.-N., He X.-S., Wang J.-R., Liu Y.-X., Wang Y., Yang X.-M., Zhang Y.-J., Gan W.-J. (2021). The Role of TGF-β Signaling Pathways in Cancer and Its Potential as a Therapeutic Target. Evid.-Based Complement. Altern. Med..

[70] Pozo F., Tenorio A. (1999). Detection and Typing of Lymphotropic Herpesviruses by Multiplex Polymerase Chain Reaction. J. Virol. Methods.

[71] Stevens S.J.C., Verkuijlen S.A.W.M., Hariwiyanto B., Harijadi, Fachiroh J., Paramita D.K., Tan I.B., Haryana S.M., Middeldorp J.M. (2005). Diagnostic Value of Measuring Epstein-Barr Virus (EBV) DNA Load and Carcinoma-Specific Viral mRNA in Relation to Anti-EBV Immunoglobulin A (IgA) and IgG Antibody Levels in Blood of Nasopharyngeal Carcinoma Patients from Indonesia. J. Clin. Microbiol..

[72] Conway C., Graham J.L., Chengot P., Daly C., Chalkley R., Ross L., Droop A., Rabbitts P., Stead L.F. (2015). Elucidating Drivers of Oral Epithelial Dysplasia Formation and Malignant Transformation to Cancer Using RNAseq. Oncotarget.

[73] Afgan E., Baker D., Batut B., van den Beek M., Bouvier D., Čech M., Chilton J., Clements D., Coraor N., Grüning B.A. (2018). The Galaxy Platform for Accessible, Reproducible and Collaborative Biomedical Analyses: 2018 Update. Nucleic Acids Res..

[74] Volders P.-J., Anckaert J., Verheggen K., Nuytens J., Martens L., Mestdagh P., Vandesompele J. (2019). LNCipedia 5: Towards a Reference Set of Human Long Non-Coding RNAs. Nucleic Acids Res..

[75] Li Y., Andrade J. (2017). DEApp: An Interactive Web Interface for Differential Expression Analysis of next Generation Sequence Data. Source Code Biol. Med..

[76] Tang D., Chen M., Huang X., Zhang G., Zeng L., Zhang G., Wu S., Wang Y. (2023). SRplot: A Free Online Platform for Data Visualization and Graphing. PLoS ONE.

[77] Wickham H. (2016). Ggplot2: Elegant Graphics for Data Analysis.

[78] Livak K.J., Schmittgen T.D. (2001). Analysis of Relative Gene Expression Data Using Real-Time Quantitative PCR and the 2−ΔΔCT Method. Methods.

[79] Ye J., Coulouris G., Zaretskaya I., Cutcutache I., Rozen S., Madden T.L. (2012). Primer-BLAST: A Tool to Design Target-Specific Primers for Polymerase Chain Reaction. BMC Bioinform..

[80] The Cancer Genome Atlas Network (2015). Comprehensive Genomic Characterization of Head and Neck Squamous Cell Carcinomas. Nature.

[81] Vlachos I.S., Zagganas K., Paraskevopoulou M.D., Georgakilas G., Karagkouni D., Vergoulis T., Dalamagas T., Hatzigeorgiou A.G. (2015). DIANA-miRPath v3.0: Deciphering microRNA Function with Experimental Support. Nucleic Acids Res..

[82] Chen Y., Wang X. (2020). miRDB: An Online Database for Prediction of Functional microRNA Targets. Nucleic Acids Res..

[83] Liu W., Wang X. (2019). Prediction of Functional microRNA Targets by Integrative Modeling of microRNA Binding and Target Expression Data. Genome Biol..

[84] Shirdel E.A., Xie W., Mak T.W., Jurisica I. (2011). NAViGaTing the Micronome—Using Multiple MicroRNA Prediction Databases to Identify Signalling Pathway-Associated MicroRNAs. PLoS ONE.

[85] Tokar T., Pastrello C., Rossos A.E.M., Abovsky M., Hauschild A.-C., Tsay M., Lu R., Jurisica I. (2018). mirDIP 4.1—Integrative Database of Human microRNA Target Predictions. Nucleic Acids Res..

[86] Huang H.-Y., Lin Y.-C.-D., Cui S., Huang Y., Tang Y., Xu J., Bao J., Li Y., Wen J., Zuo H. (2022). miRTarBase Update 2022: An Informative Resource for Experimentally Validated miRNA–Target Interactions. Nucleic Acids Res..

[87] Agarwal V., Bell G.W., Nam J.-W., Bartel D.P. (2015). Predicting Effective microRNA Target Sites in Mammalian mRNAs. eLife.

[88] Paz I., Kosti I., Ares M., Cline M., Mandel-Gutfreund Y. (2014). RBPmap: A Web Server for Mapping Binding Sites of RNA-Binding Proteins. Nucleic Acids Res..

[89] Kang J., Tang Q., He J., Li L., Yang N., Yu S., Wang M., Zhang Y., Lin J., Cui T. (2022). RNAInter v4.0: RNA Interactome Repository with Redefined Confidence Scoring System and Improved Accessibility. Nucleic Acids Res..

[90] Shannon P., Markiel A., Ozier O., Baliga N.S., Wang J.T., Ramage D., Amin N., Schwikowski B., Ideker T. (2003). Cytoscape: A Software Environment for Integrated Models of Biomolecular Interaction Networks. Genome Res..

[91] Mi H., Thomas P., Nikolsky Y., Bryant J. (2009). PANTHER Pathway: An Ontology-Based Pathway Database Coupled with Data Analysis Tools. Protein Networks and Pathway Analysis.

[92] Schneider C.A., Rasband W.S., Eliceiri K.W. (2012). NIH Image to ImageJ: 25 Years of Image Analysis. Nat. Methods.

