# Supplementary material for: The Oncogenic Role of VWA8-AS1, a Long Non-Coding RNA, in Epstein–Barr Virus-Associated Oral Squamous Cell Carcinoma: An Integrative Transcriptome and Functional Analysis
Source: Int J Mol Sci. 2024 Nov 22;25(23):12565. doi: 10.3390/ijms252312565 (PMC11641457; doi:10.3390/ijms252312565)
Supplement: Supplementary file 1 [file ijms-25-12565-s001.zip › Table S1_Selected OSCC RNA-seq Dataset Sample ID .pdf]

Table S1. Selected OSCC RNA-seq Dataset Sample ID.

Sample	Sample accession number	Experiment accession number	Sample	Sample accession number	Experiment accession number
PG004-N	SAMEA2804151	ERX605413	PG004-T	SAMEA2804189	ERX605415
PG038-N	SAMEA2804136	ERX605389	PG137-T	SAMEA2804180	ERX605394
PG049-N	SAMEA2804138	ERX605376	PG063-T	SAMEA2804177	ERX605378
PG063-N	SAMEA2804139	ERX605380	PG038-T	SAMEA2804174	ERX605379
PG079-N	SAMEA2804152	ERX605416	PG079-T	SAMEA2804190	ERX605419
PG086-N	SAMEA2804149	ERX605388	PG086-T	SAMEA2804187	ERX605390
PG105-N	SAMEA2804137	ERX605407	PG174-T	SAMEA2804185	ERX605408
PG108-N	SAMEA2804140	ERX605383	PG108-T	SAMEA2804178	ERX605381
PG122-N	SAMEA2804153	ERX605423	PG122-T	SAMEA2804191	ERX605425
PG123-N	SAMEA2804146	ERX605397	PG123-T	SAMEA2804184	ERX605398
PG129-N	SAMEA2804141	ERX605384	PG129-T	SAMEA2804179	ERX605386
PG136-N	SAMEA2804145	ERX605373	PG049-T	SAMEA2804176	ERX605374
PG137-N	SAMEA2804142	ERX605392	PG136-T	SAMEA2804183	ERX605396
PG144-N	SAMEA2804143	ERX605400	PG105-T	SAMEA2804175	ERX605399
PG146-N	SAMEA2804144	ERX605403	PG144-T	SAMEA2804181	ERX605402
PG174-N	SAMEA2804147	ERX605406	PG146-T	SAMEA2804182	ERX605405
PG187-N	SAMEA2804150	ERX605412	PG187-T	SAMEA2804188	ERX605410
PG192-N	SAMEA2804148	ERX605420	PG192-T	SAMEA2804186	ERX605422

***N:** Normal Adjacent Tissues, T; Tumor Tissues
